# Cocaine in Diarrhoea

**Published:** 1913-06

**Authors:** E. Fuld

**Affiliations:** Berlin


**Cocaine in Diarrhoea.**

Berlin, April 14, 1913.

Esteemed Colleague:

. . . . At present it is impossible to prepare an article for the BUFFALO MEDICAL JOURNAL on account of pressure of other work, but I hope to have that pleasure later. You may have noticed my method of treating diarrhoea with local anaesthetics (*Semaine Medicale,* 1912), corroborated by at least two other writers. The long and short of it is that one gives 10 drops of a 3% cocaine solution, in water, three times a day, before meals—or other local anaesthetics may be used. The practical value of the method lies in the fact that no special diet is required, that no habit is engendered, and that neither obstipation nor other untoward after-effect is produced. . . . . . I have had no experience with and know of no reference in the literature to such a case as you mention. (Ascites, elephantiasis of both lower limbs, sharply demarcated abdominal wall anasarca with prominent lymphatic channels, reported by the editor, subsequently shown by necropsy to be due to tumor near receptaculum.)

With best wishes, E. FULD.

